# Employee strategic goal sight and strategic action: the moderating role of openness to experience

**DOI:** 10.3389/fpsyg.2025.1434575

**Published:** 2025-04-28

**Authors:** Feng Hu, Xiao Jie Lu, Zeng qing Wei, Juan Peng, Shichang Liang, Zhi xuan Gao

**Affiliations:** ^1^School of Business, Guangxi University, Nanning, China; ^2^Talents Service Center of Guangxi Zhuang Autonomous Region, Nanning, China

**Keywords:** employee strategic goal sight, strategic action, perceived insider status, openness to experience, strategic development

## Abstract

**Introduction:**

Scholars have examined various factors influencing employee actions, such as goal congruence, personality traits, and job fit. However, they have overlooked employees’ strategic goal sight. This paper investigates how employees strategic goal sight affects their strategic actions and explores the moderating influence of their openness to experience.

**Methods:**

A questionnaire survey of 908 employees from various companies was conducted, and data analysis was performed using AMOS and SPSS.

**Results:**

(1) Employee strategic goal sight significantly influences employee strategic actions positively; (2) Perceived insider status acts as a mediator between employee strategic goal sight and employee strategic actions; and (3) Openness to experience moderates this effect, as evidenced by: With increasing levels of employees openness to experience, the positive impact of their strategic goal sight on perceived insider status and strategic actions gradually diminishes.

**Discussion:**

These findings not only enhance understanding of the relationship between employees’ strategic goal sight and strategy but also offer significant implications for guiding employees to engage in strategic behaviors that foster the company’s strategic development.

## 1 Introduction

In today’s competitive and ever-changing business environment, the success of organizations often depends on the accuracy of organizational strategies and the motivation of employee involvement (Sawe et al., 2021). As important stakeholders of a company, employees are critical to the success of a company as they are directly involved in the implementation of strategy; therefore, the implementation of employee strategic actions is key to the survival and growth of the organization (Wang et al., 2023). However, many organizations face the challenge of implementing strategy by eliminating silo thinking and developing a common understanding of strategy (Porck et al., 2020), and how employees, as the main implementers in the process of corporate strategy implementation, understand and embrace the organization’s strategic goals, and how they translate this understanding into concrete actions, is an important factor in ensuring the organization’s success in the long term (Köseoglu et al., 2020; Engert and Baumgartner, 2016). Employee strategic goal sight responds to the extent to which employees understand and agree with the strategic goals of the organization, which determines their behavior and performance in the firm (Boswell, 2006), and therefore it is important to examine the impact of employee strategic goal sight on employee strategic actions.

Prior to the formal proposition of the concept of employee strategic goal line of sight, scholars had already initiated research to investigate the influence of employees’ cognitive attitudes and behaviors on strategy. Some scholars have also examined employees’ attitudes and behaviors using pertinent human resource methods and models (Schuler and Jackson, 1987). Moreover, research indicates that when employees align their attitudes with the strategic goals of the organization, there is a high likelihood of them fully accomplishing and achieving these goals (Truss, 2001). The aforementioned research underscores the significance of employees in actualizing the strategic objectives of the enterprise, primarily emphasizing their abilities and characteristics. However, it does not ascertain whether employees possess a genuine comprehension of the enterprise’s strategic objectives or engage in strategic actions to contribute to the enterprise. Boswell (2006) introduced the concept of employee strategic goal line of sight, which centers on assessing whether individual employees comprehend the organization’s strategy and are capable of effectively contributing toward the achievement of its strategic goals.

Research on employee strategic goal sight and strategic action can be categorized into three main areas. Firstly, scholars have investigated the antecedents and consequences of employee strategic sighting. For instance, Cheyne (2021) discovered that the foresight and strategic thinking abilities of non-profit organization leaders positively influence employee strategic sighting. Buller and McEvoy (2012) proposed that employee strategic sighting contributes to the development of human and social capital, crucial for achieving and maintaining performance excellence. Lewis (2023) examined how communication fosters employee alignment with business strategy and enhances their strategic sighting. Additionally, Alfes et al. (2013) scrutinized how employees translate strategic sighting into actionable steps, shedding light on the underlying psychological and behavioral mechanisms. Despite the widespread recognition of employee strategic goal sight, there remains a dearth of empirical research addressing how employees actualize their perceptions of strategy into tangible actions. The second aspect concerns research related to perceived insider status and its impact on employee behavior. Perceived insider status refers to employees’ cognitive and emotional experiences regarding their position and role within the organization. It is considered a potential mechanism linking cognition and behavior, a concept widely acknowledged in the field of strategic management (Fan P. et al., 2023; Posey et al., 2015; Schaubroeck et al., 2017; Stamper and Masterson, 2002), However, the present study did not investigate the mediating role of perceived insider status in shaping employees’ strategic goal sights. Thirdly, research on openness to experience in action has revealed its association with innovative behavioral aspects (Hammond et al., 2011; Moss et al., 2007), Nonetheless, openness to experience is frequently recognized as a significant factor shaping individual behaviors and attitudes in organizational behavioral and psychological research, and it is considered one of the personality traits linked with strategic action (George and Zhou, 2001). Levels of experiential openness may influence individuals’ comprehension, response, and implementation of organizational strategies, consequently affecting their strategic actions. However, this perspective remains relatively under-explored in existing research.

Building on this premise, the present study conducts a comprehensive investigation into the relationship among employees’ strategic goal sights, perceived insider status, and their strategic actions. This inquiry revolves around three primary inquiries: (1) Does employee strategic goal sight directly influence employee strategic actions? (2) What mechanisms and pathways underlie the influence of employees’ strategic goal sights on their strategic actions? Specifically, does perceived insider status mediate this relationship? (3) What are the pertinent boundary conditions influencing the impact of employee strategic goal sight on employee strategic action? In other words, how do these effects vary across employees with differing levels of openness to experience?

To address these issues, the study develops a moderated mediation model based on goal congruence theory, investigating the relationship among employees’ strategic goal sight, perceived insider status, and experiential openness concerning employees’ strategic actions. The primary objective is to scrutinize how employees’ strategic goals sights mediate (via insider status perception) and moderate (through openness to experience) employees’ strategic actions. This research enhances understanding in the field of strategic management regarding the mechanisms influencing employees’ strategic actions, accounting for employees’ personality traits, and offering insights into how firms can effectively leverage their human resources to advance organizational strategic goals.

The study introduces innovations across three key dimensions. Firstly, it investigates the influence of employees’ strategic goal sight on their strategic actions, departing from prior research that mainly focused on strategic actions. This study delves into employees’ individual cognitive understanding of strategic goals, introducing the concept of strategic goal sight, which is pivotal for understanding employees’ strategic actions. Secondly, it highlights the mediating role of perceived insider status in linking employees’ strategic perceptions with their actions, enriching the understanding of the formation mechanism of employees’ strategic actions in strategic management. It elucidates how perceived insider status facilitates employees in translating their perceptions of the organization’s strategic goals into actionable steps. Lastly, it explores how individual employee differences, particularly openness to experience, influence strategic behavior and assesses variations in the impact of employees’ strategic goal sights on their strategic actions across different personality traits.

### 2 Rationale and research hypothesis

### 2.1 Literature review

#### 2.1.1 Employee strategic goal sight

Boswell (2006) defined Strategic Goal Sight (hereafter SGS) as employees’ fundamental grasp of the organization’s strategic objectives. This concept, intricately linked with goal setting, incentives, and leadership, is frequently utilized to gauge the effective dissemination and execution of a company’s strategic goals across various echelons. Expanding upon Boswell’s individual-focused line-of-sight model. Expanding upon Boswell’s individual-focused line-of-sight model, Buller and McEvoy (2012) posited that organizational performance sees enhancement when organizational competencies and culture, team competencies and norms, and employee skills, motivations, and opportunities align with the company’s strategic goals (i.e., when there exists a clear “line of sight”). This study delves into employee strategic sight, as defined by Boswell (2006), which denotes the degree to which employees’ conduct and performance align with the strategic goals and overall trajectory of the firm. The success of a well-operating organization hinges on employees’ capacity to comprehend and align themselves with its strategic goals. When a company disseminates instructions downward, mere acquiescence from employees without a profound understanding of the company’s goals leads to an inability to achieve the desired outcomes (Simon, 1991). Simon (1991) emphasizes the necessity for employees to proactively acquire knowledge about the organization to understand its needs comprehensively and utilize their skills to contribute effectively, thereby aiding in goal achievement. Lawler (1994), in the realm of human resource management practice, notes a shift in employees’ focus from task-level implementation to strategic-level work, signifying a transition toward corporate strategy awareness, indicative of employees possessing strategic goal sight.

Research demonstrates the substantial impact of employee strategic goal sight on business development. It has been found that employee strategic goal sight has a significant contribution to business development. Truss (2001) asserts that when employees’ attitudes align with the organization’s strategic goals, they are more likely to fully achieve them. Thundiyil (2015) investigated the influence of employee goal sight and behavioral alignment on corporate change, finding that they promote employee behavioral congruence, with employee goal sight facilitating behavioral alignment. Scholarly inquiry has also scrutinized factors affecting employees’ strategic goals sight. For instance, Shaw and Gupta (2015) examined the impact of incentive mechanisms and performance appraisal on this aspect, concluding that well-designed mechanisms and appraisal systems enhance the alignment of employees’ behaviors with organizational goals. Van Vianen (2018) emphasized the necessity for consistency between individuals and organizations in values, problem-solving approaches, and resource allocation, advocating alignment of companies and employees with strategic goals. Kim et al. (2020) investigated how corporate strategic alignment influences employees’ comprehension and pursuit of strategic goals, affecting organizational performance. They also noted significant variations in employees’ understanding of strategic goals across different job roles. Balzano et al. (2023) argued that employees’ comprehension of the organization’s strategic goals positively impacts firm performance and goal achievement, with variations based on employees’ experience levels.

#### 2.1.2 Employee strategic action

Employee strategic action (hereinafter referred to as ESA) refers to specific actions or initiatives taken by employees to achieve the organization’s strategic goals. Boswell (2006) introduced the concept of employee strategic vision by analyzing two perspectives from the employee’s point of view: the employee’s understanding of the firm’s current strategic goals themselves (i.e., Line of Sight-Objective); and the corresponding actions to achieve the goals (i.e., Line of Sight-Action). The control theory proposed by Tannenbaum (1962) suggested that the more employees can understand organizational goals and contribute effectively, the more they will behave in the interest of the organization. Porter and Harper (2003) suggested that timely identification of various issues arising from the organization’s strategic actions and timely adjustments in response to changes in strategic objectives and the environment are necessary. Strategic action is the process of translating strategy into organizational action to achieve strategic objectives and is a key step in the effectiveness of strategic management. Jalali (2012) emphasized that strategic actions are the main indicators of the effectiveness of strategy implementation and that having actionable, detailed, and measurable strategic actions is the key to achieving strategy success. Deci et al. (2017) argued that employees have three basic psychological needs, competence, autonomy, and relevance, the satisfaction of which promotes autonomous motivation and high-quality performance due to one’s outstanding contribution to the development of the organization’s strategic goals, which in turn triggers autonomous motivation and facilitates strategic employee action.

Some scholars have also studied the influencing factors that affect employees’ strategic actions. Heide et al. (2002) found that employee strategic goal attainment is a key factor in the translation of employees’ goals and strategies into daily actions, i.e., employee strategic actions make the formulated organizational strategies work. Abdulkadir et al. (2012) found that employee participation in the development of corporate strategy, and employee career planning promotes a genuine commitment to work that is useful for the development of the organization and influences employee strategic action. Thanyawatpornkul et al. (2016) examined the influencing factors affecting the strategic actions of FM Thailand from the perspective of the employees and found that communication, training and development, and reward recognition were the three key factors affecting the strategic actions of the employees. Alcaide-Muñoz et al. (2018) examined the impact of strategy execution and shop-floor communication on strategy implementation and found that effective communication among employees and employee involvement in strategy designation help motivate employees to take corresponding actions to achieve organizational strategy. Wäistö et al. (2024) studied the factors required for the successful implementation of the strategy, the study mainly analyzed the importance of organizational and employee-related factors for the implementation of the strategy, the study found that employee support is an important factor for the success of the organization is the achievement of the organizational strategy.

#### 2.1.3 Perceived insider status

Perceived Insider Status (PIS) refers to an employee’s cognitive and emotional understanding of their position and role within the organization, signifying a sense of belonging and identification with the organization (Stamper and Masterson, 2002). Masterson and Stamper (2003) viewed it as an employee’s perception of their insider status, encompassing access to internal space, status, and identity. Xiong Chen and Aryee (2007) proposed that employees’ perception of being “insiders” may reflect the cognitive aspect of self-concept, alongside its impact on identity. PIS correlates with personal job satisfaction, organizational loyalty, and alignment with the company’s strategic goals. Strong feelings of importance and belonging lead employees to prioritize organizational interests over personal ones, showing a proclivity toward centralism. Guo and Wang (2022) observed that employees with a strong PIS tend to view the company as their home, approach their responsibilities with a familial attitude, demonstrate pro-organizational behaviors, and align closely with the company’s objectives.

Wang and Kim (2013) argued that higher levels of perceived insider status are associated with stronger psychological motivations, such as a sense of belonging, leading to increased proactive behavior. Hui et al. (2015) investigated the mediating role of perceived insider status between organizational inducements and employees’ organizational citizenship behaviors. They discovered that elevated perceived insider status promotes employees’ motivation to contribute to the firm and engage in behaviors beneficial to it. Fan P. et al. (2023) examined how improper tasks can trigger withdrawal behavior by undermining perceived insider status, highlighting the impact of unreasonable tasks on employees’ perception of belonging within the organization. Supervisors and organizational support significantly influence employees’ perceived insider status. Schaubroeck et al. (2017) identified that superiors’ leadership style significantly shapes employees’ perceived insider identity. Variations in employees’ behavior may also arise based on their levels of insider identity within the organization. Proactive employee behavior has emerged as a critical factor for organizational success. Fan C. et al. (2023) explored the link between family-supportive leadership behaviors and employee proactive behaviors, revealing that perceived insider status mediates this relationship. Furthermore, they found that family-supportive leadership behaviors contribute positively to employees’ perceived insider status.

#### 2.1.4 Openness to experience

Openness to Experience (OTE) is the extent to which an individual exhibits openness, curiosity, vivid imagination, and creative thinking (Du et al., 2019). This openness extends beyond spiritual realms, encompassing the vitality, depth, originality, and complexity of real-life experiences (John and Srivastava, 1999). Characteristics of openness to experience encompass imagination, quick thinking, intellectual acumen, curiosity, artistic sensitivity, and motivation (DeYoung et al., 2007). As a facet of the Big Five personality model, openness to experience encompasses open-mindedness, curiosity, imagination, and receptivity to non-traditional viewpoints (Javed et al., 2020; Madrid et al., 2014). Highly open individuals actively seek new information and knowledge, fostering idea development from diverse knowledge sources (Schilpzand et al., 2011; Zhang et al., 2020), leading to confidence in their ideas and reduced susceptibility to authoritarian influences or biases (Madrid et al., 2014). Conversely, individuals low in openness tend to be more conservative, favoring traditional over unique ideas (George and Zhou, 2001).

Openness to experience significantly influences strategic action (George and Zhou, 2001), acting as both a predictor and a moderator of behavior (McCrae and John, 1992). Traditionally, high openness to experience is associated with a positive moderating effect, indicating a propensity for creativity and openness to new ideas in the workplace (Zhou et al., 2017), Consequently, individuals with high openness to experience are more likely to initiate innovative actions, especially in challenging situations (Cohen et al., 2019). Moss et al. (2007) noted that leaders’ trust and emotional support are particularly beneficial for fostering innovation among such employees. Similarly, Hammond et al. (2011) found a significant positive correlation between employees’ openness to experience, divergent thinking, and creative behavior. However, some studies have reported a negative moderating effect of openness to experience. For instance, Jiang (2024) observed that individuals high in openness to experience tend to cope better with work-related stressors, reducing their likelihood of immediate entrepreneurship due to enhanced adaptability and resilience in the workplace. In summary, individuals with high levels of experiential openness are exposed to diverse perspectives and ideas, whereas those lacking in experiential openness may exhibit a preference for familiarity, potentially hindering their ability to leverage motivation for learning and creativity.

### 2.2 Hypothetical derivation

#### 2.2.1 Employee strategic goal sight and employee strategic actions

By aligning the organization’s overarching direction and goals with those of individual employees, the enterprise can assess the alignment of their tasks, objectives, and career trajectories with organizational requirements. This alignment fosters individual employee performance and prioritizes tasks in accordance with organizational goals (Ayers, 2015). Simultaneously, this alignment enhances employee engagement and self-regulation, fostering appropriate employee behavior (Alagaraja and Shuck, 2015). Secondly, providing employees with a clear line of sight enables them to comprehend the company’s expectations and the significance of their contributions, fostering a strong sense of indispensability (Swarnalatha and Prasanna, 2013), This, in turn, fulfills employees’ ego needs, motivating greater engagement in their work. Finally, as employees comprehend and internalize the company’s goals, they recognize the criticality of their work in achieving these objectives, thereby enhancing motivation for greater effort and, consequently, improving performance (Shahzadi et al., 2014). This leads to the conclusion that employee goal sight has a positive effect on employee actions.

Employee strategic goal sight, reflecting employees’ comprehension of the organization’s strategic objectives, serves as the foundation for their involvement in executing the organizational strategy. Within organizations, strong alignment with the firm’s strategic objectives prompts employees to proactively align their actions to support and realize organizational strategic goals (Muafi et al., 2019). Social identity theory posits that individuals’ identification with their group heightens their motivation to uphold the group’s norms and values (Chen et al., 2016). In organizational contexts, employees who clearly identify with the firm’s strategic objectives perceive these goals as integral to their own identity, thereby strengthening their commitment and support for them. Consequently, employee strategic goal sight heightens employees’ identification with the organization, subsequently boosting their motivation to adhere to organizational norms and values (Heere and James, 2007). Secondly, according to social identity theory, individuals with a heightened sense of identity are predisposed to collaborate with fellow group members to attain shared objectives. In corporate settings, this suggests that employees possessing a strong strategic vision are likelier to collaborate with peers, fostering effective teamwork to achieve strategic objectives. Therefore, the study concludes that employees’ strategic goal sight significantly influences their strategic actions, leading to the formulation of H1.

*H1*: Employee strategic goal sight has a significant positive effect on employee strategic actions.

When employees clearly understand an organization’s strategic goals and align them with their personal goals, they are more likely to feel integral to and develop a strong sense of belonging in the organization (Davenport and Daellenbach, 2011). Employees’ perceived insider status is a deep identification with the organization and a strong sense of belonging (Stamper and Masterson, 2002), influenced by their awareness of strategic goals. Increased awareness of strategic goals helps employees feel aligned with the organization’s objectives, enhancing their perceived insider status. Moreover, employees’ connection to the organization deepens if their vision of strategic goals aligns with the organization’s values and cultural philosophy. When employees’ values and culture resonate with the organization, they identify more with it, contributing to the formation of an insider identity. Employees’ enhanced understanding of strategic goals helps them identify with the organization’s long-term and immediate goals, strengthening their sense of identification and insider identity.

Employee perceptions and attitudes are critical to organizational success and contribute to active participation in work (Harter et al., 2010). When employees feel part of the organization and resonate with its goals and mission, it significantly increases their motivation to achieve those goals (Kim et al., 2018). This perception not only highlights the inclusiveness of the organization but also reflects employees’ identification with its ethos, which becomes a key psychological variable measuring their attitudes and behaviors (Xiong Chen and Aryee, 2007). Perceived insider status is closely related to employees’ job satisfaction and organizational loyalty (Tharenou and Kulik, 2020), and is directly linked to their understanding and identification with the organization’s strategic goals. When employees strongly perceive their importance and position in the organization, they are more likely to integrate its strategic goals into their personal goals (Posey et al., 2015), leading to attitudes and behaviors aligned with these goals. Social identity theory suggests that employees develop strategic goal insight by being aware of and identifying with the organization’s strategic goals, mission, and values. Deep understanding and identification with these goals lead employees to perceive themselves as integral to the organization, fostering a sense of insider identity.

This link between identification and perception suggests a causal relationship between goal awareness and perceived insider status. Moreover, perceived insider status motivates employees to take strategic actions that align with the organization’s success. This perception instills a sense of responsibility and pride among employees, prompting them to work harder to achieve organizational goals (Xiong Chen and Aryee, 2007). Simultaneously, employees who perceive the trust and support of the organization, especially those with a high level of perceived insider status, are more likely to take actions to optimize work outcomes and contribute to the organization (Kim et al., 2019). Consequently, perceived insider status acts as a key motivator between employees’ awareness of strategic goals and their strategic actions. Employees with perceived insider status naturally exhibit positive behavioral patterns and wholeheartedly contribute to the organization’s success, driven by their deep connection to the organization and alignment with its destiny. Thus, the study proposes H2 and H3.

*H2*: There is a significant positive relationship between employees’ strategic goal sight and insider status perception.

*H3***:** perceived insider status mediates between employees’ strategic goal sights and employees’ strategic actions.

#### 2.2.2 The moderating role of openness to experience

There is a significant correlation between individual employees’ personality traits and their behavior in the organization (Mount et al., 2006). For instance, personality traits like openness, extroversion, and agreeableness may be correlated with employee performance, teamwork, and innovative behavior (Hammond et al., 2011; Jiang, 2024). Openness to experience, one of the Big Five personality traits, primarily reflects employees’ psychological adaptability and behavioral responses to change and new situations (Ashton and Lee, 2025; Srivastava et al., 2015). Moreover, high openness to experience indicates that employees are inclined to explore new areas and consider issues from diverse perspectives (McCrae and John, 1992). Employees with high openness to experience may be interested in multiple ideas and options, leading to an unstable focus and more divergent thinking (Chuang and Hsu, 2025). This may make it difficult for them to focus on understanding and exploring the organization’s strategic goals in depth, thus affecting their level of understanding and acceptance of the strategy. In contrast, employees with low openness to experience are relatively traditional and non-analytical (Desimoni and Leone, 2014); they usually do not question authority and are task-dependent employees. As a result, they are more likely to accept strategic decisions and guidance from the organization (Nyatsanga et al., 2025), which in turn affects the implementation of strategic actions. Given the diverse personalities, ages, educational backgrounds, and work experiences of corporate employees, individual employees’ openness to experience varies. Therefore, this study suggests that employees’ openness to experience negatively moderates the effect of employees’ awareness of strategic goals on their strategic actions.

Employees’ openness to experience affects employee perception (Habiboğlu et al., 2024). Individuals with high openness to experience tend to value personal autonomy and independence (Kaufman et al., 2016). Their social identities are less dependent on identification with a single group characteristic or organizational goal. They seek and build identities in multiple social groups, attenuating the impact of achieving a single organizational strategic goal on their perceived insider status. In other words, employees high in openness to experience may not significantly change their perceptions of their role in the organization due to achieving the organization’s strategic goals. Additionally, employees with high openness to experience have broad perspectives and diverse values (Christensen et al., 2019). Their openness to new ideas and approaches as well as their willingness to interact with people with different backgrounds and perspectives leads them to hold a more independent and autonomous attitude toward the achievement of the organization’s strategic goals (Habiboğlu et al., 2024), which results in a relatively weaker effect of strategic goal achievement on their insider’s perceived identity. Therefore, the study proposes H4.

*H4*: Openness to experience negatively moderates the effect of employees’ strategic goal sight on employees’ strategic actions.

*H4a*: The positive correlation between employee strategic goal sight on employee strategic actions will be diminished when employees have high openness to experience;

*H4b*: The mediating effect of perceived insider status is also attenuated when employees have high openness to experience.

The model is illustrated in Supplementary Figure 1.

## 3 Research methodology

### 3.1 Research sample

The data for this study were collected between September 6 and September 10, 2023. The questionnaire was completed by employees from various enterprises, primarily from research and development, management, and production positions, representing different job levels. We used random sampling, on the one hand, we recruited participants through the cooperation of enterprises, we directly distributed the questionnaire to the cooperative enterprises, so that the enterprises call for the participation of their employees; on the other hand, we invited participants through the online recruitment, and we gave the participants a certain amount of remuneration subsidies. This diverse sample enhances the external validity of the study. A total of 908 participants were recruited for the study and 908 questionnaires were returned. After excluding the questionnaires with identical content, answers that were not clearly differentiated, and answers that were not logical, the total number of questionnaires was 757, with a validity rate of 83.37%, and the sample for the study was 757 questionnaires.

### 3.2 Measuring tools

A Likert scale is a common survey instrument used to measure individuals’ attitudes, opinions, or beliefs about a statement or viewpoint. It is often employed in questionnaires and data collection, presenting a series of statements for individuals to rate based on their thoughts or feelings (Joshi et al., 2015). When analyzing data, Likert scales can be treated as ordered variables suitable for statistical analysis and descriptive statistics. Researchers typically calculate means, standard deviations, or other statistical indicators to summarize participants’ overall perceptions of different statements and use charts or graphs to visualize the results. Moreover, Likert scales can serve as inputs to statistical models like regression analysis, offering a quantitative basis for research.

The main variables in this study were measured using a 5-point Likert-type scale ranging from 1-completely inconsistent to 5-completely consistent. In this type of scale, each number represents a different level of intensity or degree and is usually used to measure the degree to which the subject agrees with a statement. 1-Completely inconsistent, indicating that the subject completely disagrees or strongly disagrees with the content of the statement, showing a clear negative attitude; 2-Largely inconsistent, indicating that the subject disagrees with the statement to some extent, but not as strongly as “Completely Inconsistent”; 3-Neutral, indicating that the subject does not favor either side of the argument, and is neutral toward the statement, neither favoring nor opposing it; 4-Largely consistent, indicating that the subject is neither favoring nor opposing the statement; 5-Completely Consistent, meaning that the subject agrees with or strongly agrees with the content of the statement, and shows the strongest support or agreement with it.

Employees answered questions on basic information, their strategic goal sight, strategic actions, perceived insider status, and questions related to openness to experience. Employees answer questions regarding basic information, strategic goal awareness, strategic actions, perceived insider status, and questions related to openness to experience. Basic information serves as a control variable. Based on existing scholarly research, six indicators—gender, age, education, position level, years of working experience, and team type—are selected as control variables. In order to facilitate statistical measurement, each option is assigned a value in turn, and the weighted average of the items in each sub-scale is selected as the score of the indicator, e.g., the score of strategic goal sight is obtained by the weighted average of seven items. Options are detailed in Supplementary Appendix.

#### 3.2.1 Employee strategic goal sight

The line of sight (LOS) of employees’ strategic goals refers to the extent to which employees understand and align with the organization’s strategic objectives. As each organization has unique strategic goals and specific action measures, there is no single, unified LOS scale in the existing literature; instead, more targeted scales have been developed based on specific company samples. While each company will have its strategic goal and specifics of strategic actions, this does not alter the concepts’ meanings but affects only the operational aspects of quantitative studies. The development of scales for the sample firms in this study adhered strictly to generally accepted procedures in employee strategic vision research. We constructed the scale by analyzing common strategic goals and corresponding behaviors of enterprises, following the definition and measurement of strategic goal sight regarding Boswell (2006) and Delery and Doty (1996). Through interviews with top managers of the sample companies, we identified and selected items that best matched our company’s strategic goals as measurement indicators. After detailed discussion and screening, seven items were finally selected to measure employees’ strategic goal sight, including providing cost-effective products and services and promoting specialization and standardization of products and services, detailed in Supplementary Appendix A.

#### 3.2.2 Strategic staff actions

Employee Strategic Actions (ESA) refer to specific actions and initiatives taken by employees in line with the organization’s strategic goals and directions to achieve the organization’s strategic goals. These actions include a variety of behaviors exhibited by employees at work, such as performing specific tasks, participating in decision-making, suggesting improvements, cooperating, and collaborating (Pulakos and O’Leary, 2011). Referring to previous studies (Boswell, 2006; Swarnalatha and Prasanna, 2013), and combining them with the specific items of the company’s strategic objectives selected earlier, each strategic objective proposes two to four specific actions for employees that reflect the requirements of the strategic objectives. Each action item should be specific and easy to implement, while also having a general guiding significance for all employees of the company. This study lists 19 specific employee strategic action items for measuring employee strategic actions, such as reducing waste at work, and saving costs, as detailed in Supplementary Appendix A.

#### 3.2.3 Perceived insider status

Perceived insider status (PIS) is the subjective perception of recognition and status within an organization, reflecting whether an employee sees themselves as an “insider.” It involves the cognitive and emotional experience of one’s position, responsibility, power, and relationships within the organization. This study used a unidimensional scale with three positive and three negative scoring items developed by Stamper and Masterson (2002). This scale is not only widely used internationally (Zhang et al., 2022), but has also been validated in the Chinese context by Xiong Chen and Aryee (2007) with good reliability and validity. A 6-question scale was selected for this paper, including items such as “I feel deeply that I am part of the organization” and “My organization convinces me that I am part of it,” as detailed in Supplementary Appendix B.

#### 3.2.4 Openness to experience

Openness to Experience (OTE) reflects an individual’s open-mindedness, curiosity, imagination, and originality. McCrae and Terracciano (2005) revised the NEO personality scale, it includes six main areas: aesthetics, actions, ideas, values, fantasies, and feelings, measured using questions like “I am not interested in art” and “I like the old-fashioned way I am used to.” Unlike the NEO Personality Inventory, which is limited to adolescents, the NEO-PI-3 applies to high school and college samples and may have broader applicability to adults as well Samuel et al. (2023). To avoid age-related influences on measuring openness to experience, this study used the Denissen et al. (2008) measure, which can be used across different age groups without substantial changes in the factor structure. Seven question items were used to measure openness to experience, such as “I like to think and play with ideas” and “I consider myself creative,” as detailed in Supplementary Appendix B.

## 4 Analysis of data results

### 4.1 Reliability and validity tests of the questionnaire

To ensure the questionnaire’s quality and reliability and enhance the study’s scientific validity and credibility, the analysis first assessed the reliability of four main variables: employees’ strategic goal sight, employees’ strategic actions, perceived insider status, and openness to experience. Using Cronbach’s Alpha coefficient for testing and reliability analysis in SPSS 26.0, the calculations revealed that all Cronbach’s Alpha coefficients exceeded 0.85 (SGS = 0.884, ESA = 0.981, PIS = 0.945, and OTE = 0.916), indicating high data reliability in this survey.

The study implemented several measures to control for common method bias, including anonymous measurement and partially reverse coding items, to minimize systematic bias potentially arising from participants’ questionnaire responses. Furthermore, to validate the validity and structural soundness of the measurement instrument, Meanwhile, in order to further validate the validity and structural soundness of the measurement instrument, test the hypotheses of the specific factor structure, and analyze whether the relationship between the observed and latent variables is consistent with the predefined theoretical model, we constructed a structural equation model by using Amos28 to carry out a validation factor analysis of the four variables of employees’ strategic goal sight, employees’ strategic actions, perceived insider status, and openness to experience—to assess whether the theoretical model fit the observed data.

The results in Supplementary Table 1 indicate that the four-factor model has the best fit, significantly outperforming other factor models. This demonstrates good discriminant validity, indicating that the variables are statistically distinguishable and align with the expected model structure. These findings support the study’s credibility and the instrument’s validity, showing that it accurately captures the variables and their true relationship, thereby establishing a solid foundation for the study’s results.

### 4.2 Mean, standard deviation, and correlation matrix for each variable

Descriptive statistics on the control variables of the 757 samples, the sample frequencies and percentages of each indicator are shown in Supplementary Figures 2, 3 and Supplementary Table 2. The proportion of males in the sample is significantly higher, a characteristic that may stem from a combination of factors such as industry characteristics, occupational requirements, or social structure. In addition, the proportion of employees with more than 20 years of working experience in the sample is as high as 34%, and the existence of senior employees not only brings valuable experience accumulation to the organization, but also provides a rich sample resource for our study. In terms of age structure, “post-70s” and “post-80s” employees are the main part of the sample, reflecting the age structure of the current workplace workforce. In terms of employee position level, basic employees and general managers constitute the majority of the sample, accounting for 93.39% of the total. This job level distribution feature not only reflects the broad representation of the sample in the organizational structure. The descriptive statistical analysis of the 757 sample control variables provides an important basis for our in-depth understanding of employees’ strategic goal sight, insider identity perception and strategic actions.

Firstly, we carried out a correlation analysis of the variables, which yielded a degree of freedom of the correlation coefficients of the two different variables, both of which were 755 (*df* = *755*), and the results of the correlation analyses are organized in Supplementary Table 3. A significant positive correlation was found between employee strategic goal sight and employee strategic actions (*r* = 0.681, *p* < 0.01), employee strategic goal sight and perceived insider status (*r* = 0.463, *p* < 0.01), and employee strategic goal sight and openness to experience (*r* = 0.380, *p* < 0.01). Furthermore, perceived insider status was positively correlated with openness to experience (*r* = 0.505, *p* < 0.01) and with employee strategic action (*r* = 0.619, *p* < 0.01), while openness to experience was positively correlated with employee strategic action (*r* = 0.526, *p* < 0.01). The results show a strong correlation between employee strategic goal sight, perceived insider status, openness to experience, and employee strategic action, and the existence of a high correlation between the variables provides direction for further regressions.

### 4.3 Hypothesis testing

The study was analyzed using the Process v4.1 plug-in for SPSS developed by Hayes. It examined the mediating effect of perceived insider status in the relationship between employees’ strategic goal sight and strategic actions, controlling for gender, age, education, job level, years of experience, and team type. The results are detailed in Supplementary Tables 4, 5. This approach allows for an assessment of whether perceived insider status mediates the relationship between employees’ strategic goal sight and strategic actions, considering the control variables, and thereby providing additional insight into this complex relationship.

Supplementary Table 4 indicates that introducing perceived insider status in the stepwise regression improves the explanation of employee strategic actions, justifying the inclusion of perceived insider status as a variable. In Supplementary Table 5, Model 1 shows a significant positive relationship between employee strategic goal sight and strategic actions (*B* = 0.682, *t* = 25.079, *p* < 0.001), confirming H1. With the addition of perceived insider status, the positive effect of employees’ strategic goal sight on perceived insider status was significant (*B* = 0.452, *t* = 14.2620, *p* < 0.001), establishing H2. Furthermore, there was a significant positive correlation between employees’ strategic goal sight and strategic actions (*B* = 0.503, *t* = 18.4770, *p* < 0.001), as well as between perceived insider status and strategic actions (*B* = 0.396, *t* = 14.238, *p* < 0.001), indicating that perceived insider status partially mediates the relationship, supporting H3.

To further examine the mediating effect of perceived insider status, the study decomposed the total, direct, and mediating effects between employees’ strategic goal attainment, perceived insider status, and employees’ strategic actions, as shown in Supplementary Table 6. The analysis reveals that the direct effect of employee strategic goal sight on strategic actions and the mediating effect of perceived insider status are both statistically significant, as their confidence intervals at the 95% level do not include 0 (as shown in Supplementary Table 5). This suggests that employee strategic goal sight not only directly predicts strategic actions but also does so through the mediating role of perceived insider status. After standardization, the direct effect (0.4497) and mediating effect (0.1791) accounted for 64.47 and 25.66% of the total effect (0.698), respectively.

To examine the moderating role of openness to experience in the first half of the mediator model and on the direct path, the moderated mediator model was tested while controlling for gender, age, education, job level, years of experience, and team type. The results in Supplementary Table 7 indicate that the interaction term between employees’ strategic goal sight and openness to experience was significantly associated with both employees’ strategic actions and perceived insider status (employees’ strategic actions: *B* = −0.935, *t* = 7.1971, *p* < 0.001; perceived insider status: *B* = −0.196, *t* = −4.8619, *p* < 0.001). These findings suggest that experiential openness not only moderates the positive effects of employees’ strategic goal sight on strategic actions but also moderates the positive effects of employees’ strategic goal sight on perceived insider status. Thus, H4 is supported.

Supplementary Figure 4 and Supplementary Table 8 illustrate that for subjects with lower levels of openness to experience (M-1SD), the significant positive effect of employees’ strategic goal sight on strategic actions is more pronounced. Conversely, for subjects with higher levels of openness to experience (M + 1SD), although employees’ strategic goal sight also positively influences strategic actions, the effect is smaller. This suggests that as individual openness to experience increases, the impact of employee strategic goal sight on strategic actions diminishes. The positive effect of employees’ strategic goal sight on strategic actions decreased at all three levels of empirical openness (M-1SD = 0.5164, *M* = 0.3951, M + 1SD = 0.2738).

Supplementary Figure 5 shows that for subjects with lower levels of openness to experience (M-1SD), employee strategic goal sight has a significantly positive effect on perceived insider status. Conversely, for subjects with higher levels of openness to experience (M + 1SD), employee strategic goal sight has a smaller, though still positive, effect on perceived insider status (M-1SD = 0.1077, M + 1SD = 0.0365). This indicates that the positive impact of employees’ strategic goal sight on perceived insider status diminishes as individual openness to experience increases.

Moreover, the mediating role of perceived insider status in the relationship between employee strategic goal sight and strategic actions appeared to diminish across all three levels of experiential openness. That is, as subjects’ experiential openness increased, the ability of employee strategic goal sight to stimulate strategic actions by reducing perceived insider status and thereby prompting strategic employee actions decreased. As subjects become more open to new experiences, they tend to maintain their identification with their roles and identities within the organization, seeking personal growth and autonomy. This heightened experiential openness may foster greater autonomy and independence in employees, leading them to prioritize intrinsic motivations and values over external influences. Consequently, although strategic goal sight may seek to motivate employees by shaping their perceptions of insider status, this influence may be less effective among individuals with higher levels of experiential openness.

## 5 Discussion

Research has found that employee strategic goal sight has a significant positive effect on employee strategic action. Existing studies suggest that employees’ cognitive alignment with organizational strategy is a prerequisite for fostering proactive strategic behavior (Boswell, 2006; Porck et al., 2019). Our findings corroborate this perspective by demonstrating that employees’ understanding and identification with organizational strategic goals play a pivotal role in motivating strategic actions. This reinforces the importance of social identity theory in shaping employee behavior and provides deeper theoretical insights into organizational management and strategy implementation. While prior research has linked perceived insider status to proactive behavior (Fan P. et al., 2023; Wang and Kim, 2013), our study extends this understanding by examining its impact on strategic actions. Consistent with previous findings (Stamper and Masterson, 2002; Xiong Chen and Aryee, 2007), our results indicate that a higher perceived insider status enhances employees’ engagement in strategic initiatives that contribute to organizational growth. This suggests that employees who strongly identify with their organization’s strategic goals are more likely to engage in strategic actions due to an enhanced sense of belonging and commitment.

Additionally, our study explores the moderating role of openness to experience in the relationship between strategic goal sight and strategic action, contributing to the growing body of research on personality traits in workplace behavior. While individuals with high openness to experience are generally more adaptable and proactive (Cohen et al., 2019; Zhou et al., 2017), our findings reveal a nuanced effect—high openness to experience weakens the positive relationship between strategic goal sight and strategic action. This suggests that employees with high openness to experience may face greater decision-making challenges when confronted with diverse strategic choices, thereby dampening the motivational influence of strategic goal sight. This finding aligns with prior discussions on the complexities of openness to experience in organizational settings Denissen et al. (2008) and Jiang (2024) and challenges the prevailing assumption that high openness to experience universally enhances innovation and strategic engagement. Moreover, cultural factors may moderate the relationship between openness to experience and strategic behavior across diverse organizational contexts (Chuang and Hsu, 2025; Taras et al., 2010). For instance, in highly collectivist cultures, employees scoring high on openness to experience often face tension between innovative impulses and maintaining group harmony (Williams et al., 2009), potentially resulting in less decisive strategic decision-making. Conversely, in low-power-distance cultures that value autonomy, highly open employees demonstrate greater propensity for exploratory strategic initiatives. These findings align with cross-cultural organizational studies (Akpa et al., 2021; Kirkman et al., 2017), indicating that the interplay between personality traits and cultural dimensions warrants further investigation.

By integrating these insights, our study enriches theoretical discussions on employee strategic behavior and offers practical implications for organizations seeking to optimize strategic goal communication and employee engagement strategies.

## 6 Conclusion

### 6.1 Conclusion and theoretical contributions

Employees’ perceptions of corporate goals significantly affect employee engagement, work attitudes, and behaviors, as well as job performance (Balzano et al., 2023; Thundiyil, 2015; Truss, 2001). However, few studies have explored the influence of employees’ strategic goal sight on their strategic actions. This study analyzed a questionnaire to investigate how employees’ strategic goal sight influences their strategic actions. The results demonstrate a significant positive effect of employees’ strategic goal sight on their strategic actions. Further analysis reveals that perceived insider status plays a mediating role between employees’ strategic goal sight and their strategic actions. This highlights the importance of employees’ identification with and belonging to the organization in translating strategic goals into practical actions, enriches the literature on this topic in employee behavior research, fills a gap in the literature on factors influencing employee behavior, and contributes to our understanding of the complex relationship between employee perceptions and strategy execution. Openness to experience significantly moderates this mediation process. Particularly, when employees have low openness to experience, the positive effect of strategic goal sight on their strategic actions is stronger, as is the mediating effect of perceived insider status. This finding suggests that an employee’s openness to personal experience affects their perception of the organization’s internal identity and how they respond to strategic goals, thereby influencing their strategic behavior. This finding adds to the literature on employee personality traits in the field of strategic employee behavior.

### 6.2 Management insights

This study offers targeted management recommendations for organizations:

Companies should enhance employees’ understanding and identification with the organization’s strategic goals. Managers need to focus on improving employees’ understanding and recognition of the organization’s strategic objectives. First, companies should ensure that strategic objectives are clear, well-defined, and communicated to all employees. This can be achieved through regular meetings, internal circulars, and other communication channels to make employees aware of the organization’s vision, mission, and strategic direction. Second, organizations can enhance employees’ understanding of strategic objectives through training and educational activities, including strategic planning, market trends, competitive analysis, and skills training related to strategic objectives. Finally, organizations should create an environment that encourages employees to ask questions and discuss strategic objectives, enabling them to directly link their work with the organization’s strategy and improve their understanding of strategic objectives. These measures help companies establish a common strategic vision and motivate employees to actively engage in actions to achieve the organization’s strategic goals.

Given the mediating role of perceived insider status between employees’ strategic goal sight and strategic actions, managers should enhance employees’ perceptions. This includes recognizing and rewarding behaviors aligned with the organization’s strategic goals, providing career development opportunities, and creating an inclusive and respectful work environment. Establishing a positive organizational culture and work environment is key. Managers can shape this culture by emphasizing core values, sharing success stories, and recognizing employee contributions to enhance their identification with the organization. Additionally, providing challenging work assignments with developmental opportunities and offering a developmental path aligned with the organization’s mission and values can enhance employees’ sense of insider status. These measures make employees feel they are vital to the organization’s value creation and encourage active participation in strategic initiatives.

Managers should consider employees’ openness to experience when assigning tasks and designing work processes. This means that employees with high openness to experience should be given more opportunities for challenging and creative projects, including those involving innovation and change. They should also receive sufficient resources and support to manage information overload, stimulating their creativity and innovation. For employees with low openness to experience, providing clear guidance and a stable work environment can enhance their understanding of and participation in strategic goals. By leveraging employees’ openness to experience, managers can harness their potential to drive organizational innovation and development, effectively implementing strategic goals.

### 6.3 Research limitations and future research

Our findings contribute new insights for future research. This study examines the effects of employees’ strategic goal sight on their strategic actions and explores the mediating role of perceived insider status. While this study focuses on the direct and indirect effects of strategic goal sight on strategic actions, further research should explore its long-term and persistent effects. The factors influencing employees’ strategic actions are multifaceted. Apart from perceived insider status, factors such as employees’ career planning (Abdulkadir et al., 2012), training perceptions (Thanyawatpornkul et al., 2016), and effective communication (Alcaide-Muñoz et al., 2018) among employees also play a significant role. Therefore, future research could analyze the factors influencing employees’ strategic actions from various perspectives.

The study also examined the moderating role of openness to employee experience. Previous research has shown a significant relationship between individuals’ personality traits and their behavior in organizations (Mount et al., 2006), while personality traits contain multiple aspects such as responsibility, neuroticism, extraversion, and pleasantness (De Roeck and Farooq, 2018). Employees’ sense of responsibility has been found to influence their behavior, suggesting that future research could explore the impact of other personality traits on employees’ strategic goal achievement and strategic actions. Furthermore, the impact of employees’ strategic goal sight on their strategic actions can be influenced not only by individual employees’ personality traits but also by management’s leadership styles (Fan C. et al., 2023; Zhou et al., 2017). For example, different leadership styles, such as engaged, goal-oriented, and participative, have varying effects on employees’ perceptions and behaviors (Dulewicz and Higgs, 2005). Therefore, future research could examine various aspects, including the leadership styles of corporate management.

Lastly, the research sample comprises Chinese companies. Cultural differences between the East and the West suggest that employees in Western companies prioritize independence and freedom, whereas those in Eastern companies value connection with the company, welfare, and a sense of belonging (Chandra, 2012). At the same time, the study used an econometric approach to reveal the statistical associations between the variables, and the generality of the findings may be limited, in order to enhance the generality of the study, in the future, consideration could be given to expanding the sample to include data from more different countries, and to use experimental, longitudinal, or causal modeling techniques to explore the directionality of the relationship and the underlying causal mechanisms Future research needs to use experimental, longitudinal, or causal modeling approaches further validation of the model.

## Data Availability

The raw data supporting the conclusions of this article will be made available by the authors, without undue reservation.
