# Supplementary material for: Employee strategic goal sight and strategic action: the moderating role of openness to experience
Source: Front Psychol. 2025 Apr 28;16:1434575. doi: 10.3389/fpsyg.2025.1434575 (PMC12067481; doi:10.3389/fpsyg.2025.1434575)
Supplement: Supplementary file 1 [file Table_1.docx]

Supplementary Material

# Supplementary Figures and Tables

## Supplementary Figures

**Supplementary Figure 1.** Research model


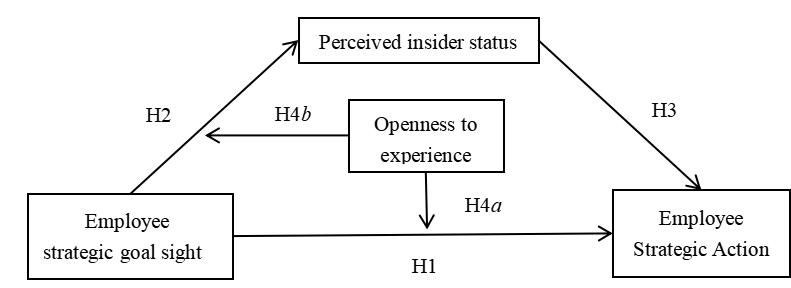


**Supplementary Figure 2.** Pie chart of sample gender distribution

**Supplementary Figure 3.** Pie chart of working years distribution

**Supplementary Figure 4.** The moderating effect of openness to experience on the relationship between employees' strategic goal sight and employees' strategic actions

**Supplementary Figure 5.** The moderating effect of openness to experience on the relationship between employees' strategic goal sight and insider's identity perception

## Supplementary Tables

**Supplementary Tables 1**. Results of confirmatory factor analysis

| Model | X^2^/df | IFI | TLI | CFI | RMSEA | SRMR |
| --- | --- | --- | --- | --- | --- | --- |
| Four-factor model  (SGS, ESA, PIS, OTE) | 2.878 | 0.963 | 0.957 | 0.963 | 0.050 | 0.042 |
| Three-factor model  (SGS+ESA, PIS, OTE) | 4.092 | 0.937 | 0.928 | 0.937 | 0.064 | 0.044 |
| Three-factor model  (SGS+PIS, ESA, OTE) | 5.955 | 0.895 | 0.885 | 0.895 | 0.081 | 0.070 |
| Three-factor model  (SGS+OTE, ESA, PIS) | 5.326 | 0.911 | 0.900 | 0.911 | 0.076 | 0.122 |
| Three-factor model  (PIS+OTE, SGS, ESA,) | 4.776 | 0.923 | 0.913 | 0.922 | 0.071 | 0.058 |
| Note: SGS represents the strategic goal sight of employees; ESA says employee strategic action; PIS represents the perception of insider identity; OTE stands for employee openness to experience. | | | | | | |

**Supplementary Table 2.** Sample distribution table

|  | Age | | Team type | | | Education | | Position Level | |
| --- | --- | --- | --- | --- | --- | --- | --- | --- | --- |
|  | Frequency | Percent | Frequency | | Percent | Frequency | Percent | Frequency | Percent |
| 1 | 61 | 8.06% | 11 | 1.45% | | 163 | 21.53% | 560 | 73.98% |
| 2 | 113 | 14.93% | 196 | 25.89% | | 74 | 9.78% | 147 | 19.42% |
| 3 | 143 | 18.89% | 432 | 57.07% | | 403 | 53.24% | 49 | 6.47% |
| 4 | 219 | 28.93% | 5 | 0.66% | | 117 | 15.46% | 1 | 0.13% |
| 5 | 221 | 29.19% | 113 | 14.93% | |  |  |  |  |
| Total | 757 | 1 | 757 | 1 | | 757 | 1 | 757 | 1 |

**Supplementary Table 3.** Mean value, standard deviation, and correlation coefficient of variables

|  | Mean value | Standard deviation | Gender | Age | Education | Position Level | Working years | Team type | SGS | ESA | PIS | OTE |
| --- | --- | --- | --- | --- | --- | --- | --- | --- | --- | --- | --- | --- |
| Gender | 1.280 | 0.448 | 1 |  |  |  |  |  |  |  |  |  |
| Age | 3.560 | 1.270 | -0.091* | 1 |  |  |  |  |  |  |  |  |
| Education | 2.630 | 0.987 | 0.109** | -0.254** | 1 |  |  |  |  |  |  |  |
| Position Level | 1.330 | 0.598 | -0.034 | 0.317** | 0.143** | 1 |  |  |  |  |  |  |
| Working years | 4.430 | 1.592 | -0.079* | 0.880** | -0.208** | 0.361** | 1 |  |  |  |  |  |
| Team type | 3.020 | 0.960 | -0.008 | 0.132** | -0.143** | -0.113** | 0.071* | 1 |  |  |  |  |
| SGS | 4.369 | 0.663 | -0.013 | -0.029 | 0.014 | 0.009 | -0.128** | -0.048 | 1 |  |  |  |
| ESA | 4.494 | 0.593 | -0.028 | -0.026 | 0.065 | 0.083* | -0.086* | 0.005 | 0.681** | 1 |  |  |
| PIS | 4.445 | 0.667 | 0.067 | -0.098** | 0.132** | 0.210** | -0.123** | -0.074* | 0.463** | 0.619** | 1 |  |
| OTE | 3.874 | 0.635 | -0.102** | -0.118** | 0.164** | 0.099** | -0.114** | -0.052 | 0.380** | 0.526** | 0.505** | 1 |
| Note: * indicates a significant correlation at 0.05 level; ** indicates a significant correlation at level 0.01. SGS stands for employee strategic vision; ESA says employee strategic action; PIS represents the perception of insider identity; OTE stands for openness to experience. The same is below. | | | | | | | | | | | | |

**Supplementary Table 4.** Results of the stepwise regression model (n=757)

| Model | | Unnormalized coefficient | | Standardization coefficient | t | Significance |
| --- | --- | --- | --- | --- | --- | --- |
|  |  | B | Standard error | Beta |  |  |
| 1 | (constant) | 1.626 | .150 |  | 10.811 | .000 |
|  | SGS | .610 | .024 | .682 | 25.079 | .000 |
|  | Gender | -.031 | .035 | -.023 | -.875 | .382 |
|  | Age | -.016 | .027 | -.034 | -.584 | .559 |
|  | Education | .028 | .017 | .046 | 1.623 | .105 |
|  | Position Level | .085 | .029 | .086 | 2.900 | .004 |
|  | Working years | .001 | .022 | .003 | .052 | .958 |
|  | Team type | .036 | .017 | .058 | 2.145 | .032 |
| 2 | (constant) | .862 | .144 |  | 5.988 | .000 |
|  | SGS | .450 | .024 | .503 | 18.477 | .000 |
|  | PIS | .352 | .025 | .396 | 14.238 | .000 |
|  | Gender | -.064 | .031 | -.048 | -2.028 | .043 |
|  | Age | .004 | .024 | .008 | .148 | .883 |
|  | Education | .017 | .015 | .028 | 1.102 | .271 |
|  | Position Level | -.015 | .027 | -.015 | -.545 | .586 |
|  | Working years | .009 | .019 | .023 | .443 | .658 |
|  | Team type | .036 | .015 | .058 | 2.403 | .017 |
| Dependent variable: ESA | | | | | | |

**Supplementary Table 5.** Test of the intermediary model of perceived insider status

| Predictor | Model 1 | | Model 2 | | Model 3 | |
| --- | --- | --- | --- | --- | --- | --- |
|  | β | t | β | t | β | t |
| SGS | 0.682 | 25.0791*** | 0.452 | 14.2620*** | 0.503 | 18.4770*** |
| PIS |  |  |  |  | 0.396 | 14.2384*** |
| R^2^ | 0.4763 | | 0.2892 | | 0.5879 | |
| F | 97.3014***_（7）_ | | 43.5302***_（7）_ | | 133.4109***_（8）_ | |
| Note: Model 1, that is, employee strategic goal sight predicts employee strategic action; Model 2, that is, employee strategic goal sight prediction perceived insider status; Model 3, that is, employees' strategic goal sight and perceived insider status jointly predict employees' strategic actions. | | | | | | |

**Supplementary Table 6.** Total effect, direct effect, and intermediate effect breakdown table

|  | Effect size | Boot standard error | 95% confidence interval | Relative effect size |
| --- | --- | --- | --- | --- |
| Total effect | 0.6098 | 0.0243 | [0.5621 0.6576] |  |
| Direct effect | 0.4497 | 0.0243 | [0.4019 0.4975] | 73.745% |
| The mediating effect of insider's identity perception | 0.1791 | 0.0305 | [0.1226 0.2418] | 29.370% |

**Supplementary Table 7.** Moderated mediation model tests

| Result variable | Predictor | Coefficient significance | Standard error | t | R | R^2^ | F(*df*） |
| --- | --- | --- | --- | --- | --- | --- | --- |
| PIS |  |  |  |  | 0.6422 | 0.4124 | 58.2551**_（9）_ |
|  | SGS | 1.038*** | 0.1518 | 6.8383 |  |  |  |
|  | OTE | 1.235*** | 0.1789 | 6.9015 |  |  |  |
|  | SGS*OTE | -0.196*** | 0.0402 | -4.8619 |  |  |  |
|  | Gender | 0.151*** | 0.0425 | 3.5448 |  |  |  |
|  | Age | 0.002 | 0.0326 | 0.0473 |  |  |  |
|  | Education | 0.004 | 0.0205 | 0.1721 |  |  |  |
|  | Position Level | 0.227*** | 0.0352 | 6.4602 |  |  |  |
|  | Working years | -0.047 | 0.0260 | -1.8203 |  |  |  |
|  | Team type | 0.001 | 0.0201 | 0.0677 |  |  |  |
| ESA |  |  |  |  | 0.7946 | 0.6314 | 127.7912**_（10）_ |
|  | SGS | 1.050*** | 0.1102 | 9.5302 |  |  |  |
|  | OTE | 0.255*** | 0.0258 | 9.8817 |  |  |  |
|  | SGS*OTE | 0.935*** | 0.1300 | 7.1971 |  |  |  |
|  | Gender | -0.170*** | 0.0288 | -5.8995 |  |  |  |
|  | Age | -0.028 | 0.0302 | -.9164 |  |  |  |
|  | Education | 0.034 | 0.0230 | 1.4775 |  |  |  |
|  | Position Level | 0.007 | 0.0145 | 0.4751 |  |  |  |
|  | Working years | -0.019 | 0.0254 | -0.7556 |  |  |  |
|  | Team type | -0.012 | 0.0184 | -0.6541 |  |  |  |

**Supplementary Table 8.** Direct and intermediate effects at different levels of openness to experience

|  | OTE | Effect size | Standard error | 95% confidence interval |
| --- | --- | --- | --- | --- |
| Direct action | 3.1429（M-1SD） | 0.5164 | 0.0291 | [0.4592 0.5736] |
|  | 3.8571（M） | 0.3951 | 0.0238 | [0.3485 0.4418] |
|  | 4.5714（M+1SD） | 0.2738 | 0.0336 | [0.2079 0.3397] |
| The mediating role of insider's identity perception | 3.1429（M-1SD） | 0.1077 | 0.0211 | [0.0709 0.1534] |
|  | 3.8571（M） | 0.0721 | 0.0188 | [0.0423 0.1152] |
|  | 4.5714（M+1SD） | 0.0365 | 0.0214 | [0.0054 0.0878] |

# Appendix

## Appendix A

| Construct | Employee Strategic Goal Sight([Boswell, 2006](#_ENREF_7); [Delery & Doty, 1996](#_ENREF_17)) | Employee Strategic Action ([Boswell, 2006](#_ENREF_7); [Swarnalatha & Prasanna, 2013](#_ENREF_64)) |
| --- | --- | --- |
| Measurement Items  (five-point Likert scale) | 1. Provide high-quality and low-priced products. | 1. Eliminate all kinds of waste and save money in the workplace.  2. Actively participate in developing and using new processes, procedures, and management tools to reduce costs.  3. To prioritize quality in production and service, and to contribute to the continuous improvement of products and services and the achievement of goals. |
|  | 2. Provide specialized and standardized products and services. | 4. Continuous improvement of work efficiency.  5. Strive to learn the rules and procedures related to the work.  6. Familiarize yourself with the operating procedures and management processes of your job.  7. Strictly comply with work rules. |
|  | 3. Customer-oriented. | 8. Thinking from the customer's point of view.  9. Strive to understand the needs and expectations of customers.  10. To achieve customer service: to be responsive, and responsive to action, action must be fruitful. |
|  | 4. Continuous research and development of new products, and continuous improvement. | 11. Keep abreast of product and technology trends in the industry.  12. Continuous research and development or support for the development of more cost-effective products. |
|  | 5. Develop new markets and realize international operations. | 13.Actively collect the latest industry information, and constantly improve their knowledge and skills.  14. Maintain good communication and cooperation with colleagues in other departments.  15. Take the initiative to share skills and knowledge and help colleagues in a timely manner. |
|  | 6. Create "China's No. 1" brand name. | 16. Actively promote the brand and values of our products to customers.  17. Build up the company's brand image by working at a high level. |
|  | 7. Transform from a product supplier to a system solution and service provider. | 18. Cooperate with customers in product design and development, and make progress together.  19. Contribute to the acceleration of digital transformation. |
| Reliability  ($\alpha$) | 0.884 | 0.981 |

## Appendix B

| Construct | Measurement Items  (five-point Likert scale) | Reliability  ($\alpha$) |
| --- | --- | --- |
| Perceived Insider Statu ([Stamper & Masterson, 2002](#_ENREF_63)) | 1. I feel deeply that I'm part of an organization. 2. My organization has convinced me that I am a part of it. 3. I feel like I'm an outsider to this organization. 4. I don't feel accepted by the organization. 5. I feel that I am an insider of the organization. 6. I feel often left out of the organization. | 0.945 |
| Openness to Experience ([Denissen et al., 2008](#_ENREF_18)) | 1. I like to think and play with ideas. 2. I feel creative. 3. I feel original and have innovative ideas. 4. I feel that I am an intelligent and deep thinker. 5. I feel I have an active imagination. 6. I am curious about many different things. 7. I prefer to work routinely. | 0.916 |
